# Impact of Maturational Status on the Ability of Osteoblasts to Enhance the Hematopoietic Function of Stem and Progenitor Cells

**DOI:** 10.1002/jbmr.302

**Published:** 2010-11-23

**Authors:** Ying-Hua Cheng, Brahmananda R Chitteti, Drew A Streicher, Joseph A Morgan, Sonia Rodriguez-Rodriguez, Nadia Carlesso, Edward F Srour, Melissa A Kacena

**Affiliations:** 1 Department of Orthopaedic Surgery, Indiana University School of MedicineIndianapolis, IN, USA; 2 Department of Medicine, Indiana University School of MedicineIndianapolis, IN, USA; 3 Department of Pediatrics, Herman B Wells Center for Pediatric Research, Indiana University School of MedicineIndianapolis, IN, USA; 4 Department of Microbiology and Immunology, Indiana University School of MedicineIndianapolis, IN, USA; 5 Department of Anatomy and Cell Biology, Indiana University School of MedicineIndianapolis, IN, USA; 6 Department of Biomedical Engineering, Indiana University Purdue University IndianapolisIndianapolis, IN, USA

**Keywords:** OSTEOBLASTS, HEMATOPOIETIC STEM CELLS, HEMATOPOIETIC NICHE, CALCIUM DEPOSITION, MOUSE

## Abstract

Osteoblasts (OBs) exert a prominent regulatory effect on hematopoietic stem cells (HSCs). We evaluated the difference in hematopoietic expansion and function in response to co-culture with OBs at various stages of development. Murine calvarial OBs were seeded directly (fresh) or cultured for 1, 2, or 3 weeks prior to seeding with 1000 Lin-Sca1 + cKit+ (LSK) cells for 1 week. Significant increases in the following hematopoietic parameters were detected when comparing co-cultures of fresh OBs to co-cultures containing OBs cultured for 1, 2, or 3 weeks: total hematopoietic cell number (up to a 3.4-fold increase), total colony forming unit (CFU) number in LSK progeny (up to an 18.1-fold increase), and percentage of Lin-Sca1+ cells (up to a 31.8-fold increase). Importantly, these studies were corroborated by in vivo reconstitution studies in which LSK cells maintained in fresh OB co-cultures supported a significantly higher level of chimerism than cells maintained in co-cultures containing 3-week OBs. Characterization of OBs cultured for 1, 2, or 3 weeks with real-time PCR and functional mineralization assays showed that OB maturation increased with culture duration but was not affected by the presence of LSK cells in culture. Linear regression analyses of multiple parameters measured in these studies show that fresh, most likely more immature OBs better promote hematopoietic expansion and function than cultured, presumably more mature OBs and suggest that the hematopoiesis-enhancing activity is mediated by cells present in fresh OB cultures de novo. © 2011 American Society for Bone and Mineral Research.

## Introduction

Recent work has expanded our understanding of the hematopoietic microenvironment in bone and the role of osteoblasts (OBs) in the regulation of hematopoietic stem cell (HSC) survival and differentiation. A hypothesis for a specialized microenvironment, or “niche,” for HSC differentiation was first posited by Schofield in 1978.(1) Shortly thereafter, a connection between bone and blood dyscrasias was suggested.(2) Although several anatomical locations of hematopoietic activity outside long bones have been ascertained(3–8) and a perisinusoidal niche for HSC differentiation has been described,(9,10) it is believed that the endosteal niche is the most important in terms of overall HSC reserve and proliferation.(11–13)

OBs have been shown to support HSC differentiation in vitro(14–16) and to play an integral role in this process in the endosteal niche.(11,17–25) OBs secrete several hematopoietic cytokines, including G-CSF, IL-1, and IL-6,(26–28) as well as osteopontin, an inhibitor of HSC differentiation.(29,30) Other endocrine mediators acting on OBs have been shown to impact differentiation and proliferation of HSCs as well. Parathyroid hormone stimulates expansion of HSC number locally in the niche and peripherally in the vasculature.(31–33) Insulin-like growth factor (IGF) has been implicated in the regulation of HSC survival and expansion.(31) It has been suggested that thrombopoietin(34) and signaling molecules from adipocytes(35) in yellow marrow also influence HSC differentiation in the niche.

A key factor in preserving the stem cell pool throughout life is the maintenance of the vast majority of HSCs at any given time in the quiescent phase of cell cycle.(36,37) OBs are critical mediators of this quiescence through intercellular signaling pathways,(13) leading to overall increased survival of HSCs.(38,39) Indeed, it has been suggested that finding quiescent HSCs in long bones is an effective means of physically identifying the endosteal niches themselves.(40) Clearly, with such complex regulatory interactions both systemically and within the niche, a thorough understanding of the complete role of the OB in the regulation of HSC survival and differentiation would be beneficial for potential clinical applications involving ex vivo manipulation of HSCs.

Most investigations using co-cultures of OBs with hematopoietic progenitor cells (HPCs) end up utilizing OBs and OB precursors in various stages of maturation and differentiation. Indeed, as Kiel and Morrison have stated,(10) most investigators call endosteal lining cells OBs, although in fact these cells are heterogeneous and include OB lineage cells at many stages of maturation, as well as other cells, including osteoclasts. Because OB lineage cells (which for convenience will be called OBs from here on) express different cell surface markers and depend on many intracellular activities as they differentiate and age,(41,42) it stands to reason that different subpopulations of OBs may behave differently with respect to regulation of HSC survival and differentiation in the niche as they age.(42) Zhu posited that a unique subpopulation of OBs may be primarily responsible for HSC support in niches.(43) We sought to discern in this study any appreciable difference in primitive hematopoietic cell expansion and function when a purified population of stem/progenitor cells, defined as Lin-Sca1 + c-kit+ (LSK) were co-cultured with OBs and OB precursors in various stages of maturation and differentiation. Using the knowledge gained from these experiments, we sought to enhance the primary function of HSCs, namely their bone-marrow-reconstituting potential, by determining that early-stage or immature OB lineage cells better promote hematopoietic activity than do more mature OB lineage cells.

## Materials and Methods

### Animals

C57BL/6 mice (2-day pups) and B6.SJL-PtγcqPep3b/BoyJ (BoyJ) mice (6 to 8 weeks old) were originally obtained from Jackson Laboratories (Bar Harbor, ME, USA). Both C57BL/6 mice and BoyJ mice were subsequently bred, housed, and used to generate F1 progeny at Indiana University. All procedures were approved by the Laboratory Animal Research Facility of the Indiana University School of Medicine and followed NIH guidelines.

### Preparation and culture of calvarial OB

Neonatal murine calvarial cells were prepared following a modification of published basic methods.(44,45) Briefly, calvariae from C57BL/6 mice less than 48 hours old were dissected out, pretreated with EDTA in PBS for 30 minutes, then subjected to sequential collagenase digestions (200 U/mL). Fractions 3 to 5 (digestions 20–35 minutes, 35–50 minutes, and 50–65 minutes) were collected and used as OBs. These cells are > 95% OBs or OB precursors as previously shown.(44,46,47) Freshly prepared OBs (0 culture duration) or OBs that were cultured for 1, 2, or 3 weeks were used for all studies.

OBs cultured for 1, 2, or 3 weeks were cultured in α-MEM supplemented with 10% FBS and with ascorbic acid (50 µg/mL added on day 0 and at all feedings) and β-glycerophosphate (5 mM added starting on day 7 and all subsequent feedings). It should be noted that these culture duration studies were set up in two different ways to control for experimental variation. In half of the studies the same starting OB population was used for fresh, 1-, 2-, and 3-week cultures, and LSK cells were generated each week from different mice. In the other half of the studies the same LSK population was used for all cultures, but the OBs were generated weekly for 4 weeks.

### LSK preparation and cell sorting

Low-density bone marrow cells were prepared from flushed long bones of BoyJ (CD45.1) mice. Cells were washed once with stain wash (PBS, 1% bovine calf serum, and 1% penicillin-streptomycin, Pen/Strep) then stained as indicated below for 15 minutes on ice. Briefly, cells were stained with phycoerythrin (PE)–conjugated lineage markers, including monoclonal antibodies (Ab) for CD3, CD4, CD45R, Ter119, and Gr1; allophycocyanin (APC)–conjugated cKit (CD117); and fluorescein isothiocyanate (FITC)–conjugated Sca1 (BD Biosciences, Rockville, MD, USA). Lin-Sca1 + cKit+ (LSK) cells were sorted on a BDIS FACS Aria (BD Biosciences).

### Co-culture of OB and LSK cells

Freshly prepared OBs or cultured OBs from C57Bl/6 mice (CD45.2) were plated at 40,000 cells per well in a 12-well plate. Freshly sorted LSK cells (1000/well) from BoyJ mice (CD45.1) were added 24 hours later. Cultures were supplemented with murine SCF and IL3 (10 ng/mL), IGF1 and TPO (20 ng/mL), IL6 and Flt3 (25 ng/mL) and osteopontin (50 ng/mL); in some experiments cytokines were not added to assess whether OBs were using or binding cytokines. Cultures were maintained for 1 week in medium consisting of a 1:1 mix of Iscove's modified Dulbecco's medium (IMDM) and α-MEM supplemented with 10% FBS, 1% Pen/Strep, and 1% L-glutamine. Cells were harvested on day 7 and counted. LSK fold increase was calculated by phenotypic analysis relative to d0 count of 1000. In each experiment, LSK cells were also seeded in wells containing no OBs as a control.

### LSK phenotyping

The entire cell content of each well was harvested and stained with all of the above antibody combinations except CD117. In addition, cells were also stained with pacific blue (PB)-conjugated CD45.1 and phycoerythrin-Cy7 (PE-Cy7)–conjugated CD45.2. CD45.1+ cells were gated and analyzed for the presence of Lin-Sca1+ cells. Data were collected on an LSRII flow cytometer (BDIS) and the percentage of Lin-Sca1+ cells determined.

### Clonogenic assays

Cultured cells or freshly sorted cells were plated in duplicate in 3-cm petri dishes containing 1 mL methylcellulose with cytokines (MethoCult GF M3434, Stem Cell Technologies, Vancouver, BC, Canada) according to the directions of the manufacturer. Either 500 freshly isolated LSK cells or a predetermined number of cultured cells (chosen to yield approximately 100 colonies per 3-cm dish) were assayed in each run. Cultures were maintained at 37 °C in humidified incubator at 5% CO_2_ and colonies were counted on an inverted microscope after 7 days. Colony types were not enumerated separately and thus a total number of clonogenic cells was reported.

### Cell cycle analysis

For the analysis of cell cycle status, co-cultured LSK progeny were collected by repeated gentle washing of the cultures to collect LSK progeny and leave adherent OBs in the plate. Collected cells were centrifuged and stained with equal volumes of staining buffer (0.1 mg/mL PI + 0.6% Nonidet P40 in PBS) and 2 mg/mL RNase as described previously.(48) Cells were triturated and incubated on ice for 30 minutes. Data were collected on an LSRII flow cytometer (BDIS) and the percentages of cells in G0/G1, S, and G2 + M phases were determined by ModFit cell cycle analysis software (Verity Software House, Topsham, ME, USA). Cell cycle status of fresh and cultured OB was determined from separate cultures that were not seeded with LSK cells.

### Apoptosis analysis

To analyze apoptosis in cultured LSK cells, co-cultured LSK progeny were collected as described above for cell cycle analysis. Collected cells were then washed once in Dulbecco's modified eagle medium (DMEM) followed by the addition of annexin V conjugated with allophycocyanin (APC). Cells were incubated on ice for 15 minutes, then washed and resuspended in DMEM followed by the addition of 10 µL of 10 ng/mL propidium iodide (PI). Cells were incubated at room temperature for an additional 10 minutes and data were collected on an LSRII flow cytometer (BDIS). The percentage of cells that were positive for annexin V and negative for PI was determined. Apoptosis of fresh and cultured OB was also determined, as above, from separate cultures that were not seeded with LSK cells.

### Quantitative real-time (RT) PCR

Total RNA was isolated using RNAeasy kit (Qiagen, Valencia, CA, USA). DNAse (Qiagen)–treated RNA was used to generate cDNAs by reverse transcription according to the manufacturer's instructions (SuperScript II kit; Invitrogen, Carlsbad, CA, USA). PCR reactions were performed in an MX3000 detection system using SYBR green PCR reagents following the manufacturer's instructions (Stratagene, La Jolla, CA, USA). For each gene analyzed (see Table 1 for primer sequences), a calibration curve was performed and all the oligonucleotides were tested to ensure specificity and sensitivity. For each sample, arbitrary units obtained using the standard curve and the expression of GAPDH was used to normalize the amount of the investigated transcript.

**Table 1:** Primer Sequences for Real-Time PRC Reactions

Runx2 forward primer	5' CGACAGTCCCAACTTCCTGT
Runx2 reverse primer	5' CGGTAACCACAGTCCCATCT
Alkaline phosphatase forward primer	5' GCTGATCATTCCCACGTTTT
Alkaline phosphatase reverse primer	5' CTGGGCCTGGTAGTTGTTGT
Osteocalcin forward primer	5' AAGCAGGAGGGCAATAAGGT
Osteocalcin reverse primer	5' TTTGTAGGCGGTCTTCAAGC
Osteopontin forward primer	5' ACTCCAATCGTCCCTACAGTCG
Osteopontin reverse primer	5' TGAGGTCCTCATCTGTGGCAT
Type I collagen forward primer	5' CAGGGAAGCCTCTTTCTCCT
Type I collagen reverse primer	5' ACGTCCTGGTGAAGTTGGTC
Jagged 1 forward primer	5' TCCAGGTCTTACCACCGAAC
Jagged 1 reverse primer	5' GGACGCCTCTGAACTCTGAC
Jagged 2 forward primer	5' GAGGTCAAGGTGGAAACAGT
Jagged 2 reverse primer	5' TGTCCACCATACGCAGATAA
Stem cell factor forward primer	5' CCTTAGGAATGACAGCAGTAG
Stem cell factor reverse primer	5' AGCCAATTACAAGCGAAATGAG
GAPDH forward primer	5' CGTGGGGCTGCCCAGAACAT
GAPDH reverse primer	5' TCTCCAGGCGGCACGTCAGA

### Quantitative analysis of calcium deposition

Calcium deposition was assessed by eluting alizarin red S from cell monolayers as previously described.(49) Briefly, monolayers were washed 2 × with PBS, subsequently fixed in ice-cold 70% (v/v) ethanol for 1 hour, then washed 2 × with water. Monolayers were stained with 40 mM alizarin red S (pH 4.2) for 10 minutes (room temperature, shaking), unbound dye was removed by washing with water (5 ×) and with PBS (1 × for 15 minutes, room temperature, shaking). Bound alizarin red was eluted by incubating monolayers with 1% (v/v) cetylpyridinium chloride in 10 mM sodium phosphate (pH 7.0) for 15 minutes (room temperature, shaking). Absorbance from aliquots was measured at 562 nm (GENios Plus, Tecan, San Jose, CA, USA), and alizarin red concentrations were calculated from measured standards (Ca/mol of dye in solution).

### Transplantation studies

In order to examine the long-term repopulating potential of in vitro cultured LSK cells, 2000 LSK cells per well in a 12-well plate were co-cultured for 5 days in the presence of freshly isolated or 3-week OBs. On day 5, the entire contents of each well were harvested and injected via the tail vein into a lethally irradiated (1050 cGy split dose, 3–4 hours apart) C57BL/6 X BoyJ F1 recipient (CD45.2/CD45.1) along with 100,000 competitor low-density bone marrow cells from BoyJ mice (CD45.1). Control mice received 2000 LSK cells (freshly isolated) and 100,000 competitor cells. To determine whether transplantation of OBs along with LSK cells facilitates engraftment, additional control mice received 2000 LSK cells (freshly isolated) and 100,000 competitor cells as well as OBs. Specifically, 40,000 freshly isolated OBs were plated for 5 days (as detailed for co-cultures of fresh OBs + LSK cells), the well was trypsinized, and OB cells were counted and injected with freshly isolated LSK and competitor cells into mice. Transplanted mice received doxycyclin-treated commercial feed pellets for 2 weeks prior to and 3 weeks after transplantation. At monthly intervals, for 4 months, chimerism in blood was assessed as CD45.2/(CD45.2 + CD45.1) × 100. This calculation was therefore independent of any residual host-derived HSC activity. The mice were euthanized at 4 months, and bones were collected and marrow flushed out of the femurs. The marrow was then stained as described above with CD45.1 and CD45.2 antibodies, and chimerism was also determined as described above.

### Statistical analyses

Unless otherwise indicated, data are presented as the mean ± the standard error associated with the mean. Student's *t* tests were performed when only two groups were compared. One-way factorial analyses of variances were used to make multiple group comparisons. Pair-wise Bonferroni comparisons were made to explore individual group differences while controlling for the elevated family-wise error associated with performing multiple comparisons. Linear regressions using analysis of variance model were performed to compare continuous data groups. All analyses were performed with Statistical Package for Social Sciences (SPSS 16; Norusis/SPSS, Chicago, IL, USA) software and were 2-tailed with a level of significance set at 0.05.

## RESULTS

### OBs enhance HPC proliferation, CFU expansion, maintenance of primitive phenotype, and plating efficiency

**Fig. 1:**
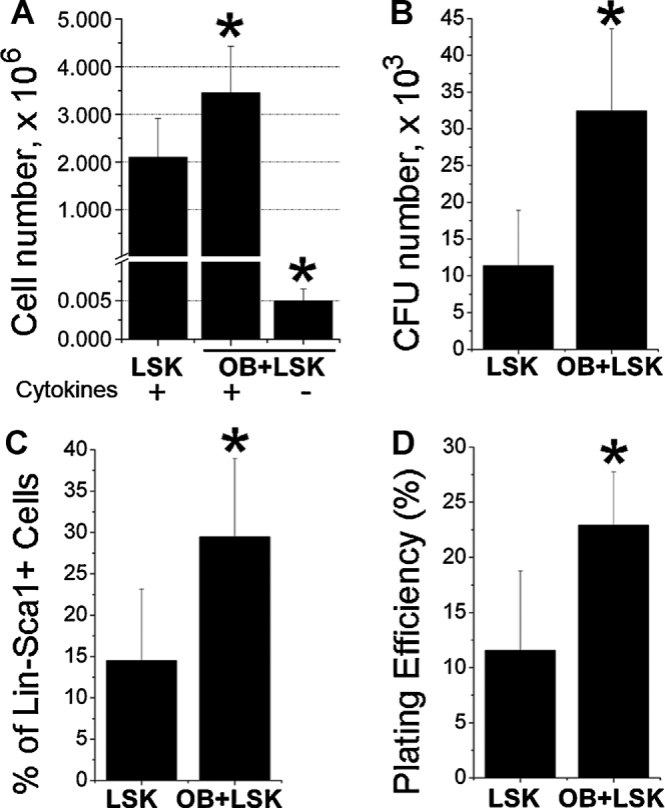
LSK cells were cultured alone or in the presence of freshly prepared OBs for 7 days and the following parameters were measured: (*A*) total number of hematopoietic cells in culture; (*B*) total number of CFU produced in culture; (*C*) percentage of cells expressing Lin-Sca1+ cell surface markers; and (*D*) plating efficiency of clonogenic cells (number of CFUs/number of cells assayed × 100). Data are presented as the mean ± the standard deviation. Co-culture of LSK cells with OBs significantly increased all hematopoietic parameters analyzed relative to LSK cells cultured alone. *Indicates statistically significant differences (*p* < .05) compared to LSK cells alone for identical HPC measurements.

It is well understood that OBs play an important role in the HSC niche. Our goal in this study was to determine whether OB maturation altered hematopoietic potential of primitive progenitor cells. However, we had to first show that in our model system, OBs enhance in vitro hematopoiesis, which is usually assessed through increased numbers of clonogenic cells and cells that maintain the original phenotype of primitive progenitors, in this case the LSK phenotype. As shown in Fig. 1, compared with LSK cells cultured alone, co-culture with OBs promoted all HPC properties tested. As illustrated in Fig. 1, LSK cells cultured alone (*n* = 6–8) versus LSK cells co-cultured with OBs (*n* = 7–9) for 7 days resulted in the following: hematopoietic cell proliferation (2.1 ± 0.8 × 10^6^ vs. 3.5 ± 1.0 × 10^6^, *p* = .008; Fig. 1*A*), production of clonogenic cells (11,400 ± 7500 vs. 32,400 ± 11,200, *p* < .001; Fig. 1*B*), percentage of Lin-Sca1+ cells (14.5 ± 8.7% vs. 29.5 ± 9.5%, *p* = .01; Fig. 1*C*), and plating efficiency (11.6 ± 7.2 vs. 22.9 ± 4.8, *p* = .002; Fig. 1*D*).

We also established co-cultures in the absence of cytokines and examined hematopoietic cell proliferation (Fig. 1*A*). While hematopoietic cells added to OBs survived for a period of 7 days in the absence of cytokine supplementation (whereas hematopoietic cells cultured without OBs and without cytokines did not survive, data not shown), the degree of expansion of these cells during this period was very limited and precluded the pursuit of characterizing the function of these cells. It should also be noted that since c-kit expression is lost(50–52) when cells are exposed to SCF, we determined the percentage of Lin-Sca1+ cells instead of Lin-Sca1 + c-kit+ cells on day 7. Supplemental Fig. 1*A* illustrates this loss of c-kit expression for Lin-Sca1+ cells. The ligand for c-kit, SCF, is expressed in OBs at the same levels regardless of culture duration (fresh OBs, 1-week OBs, and 2-week OBs, Supplemental Fig. 1*B*). Although the experiments presented here use neonatal calvarial OBs, we previously demonstrated that OBs derived from both long bones (in a manner similar to that described by Balduino et al(53)) and calvariae of neonatal and young adult mice resulted in enhancement of the same hematopoietic properties studied here.(54)

### Effects of OB culture duration on HPC proliferation, CFU expansion, maintenance of primitive phenotype, plating efficiency, cell cycle status, and apoptosis

**Fig. 2:**
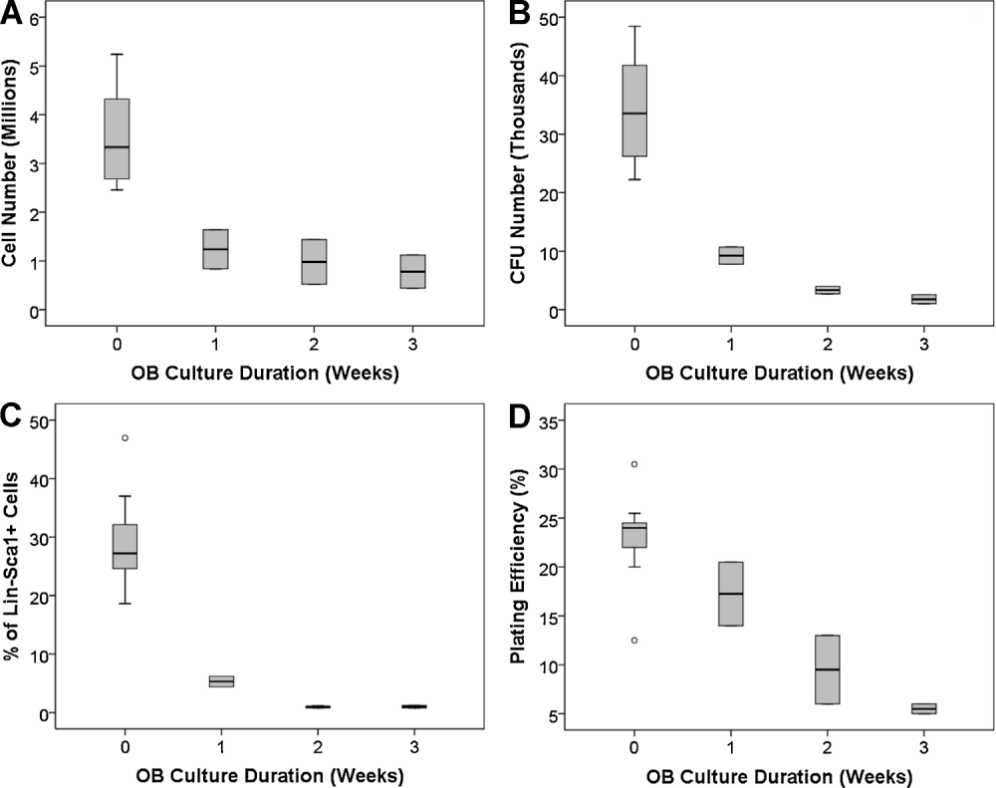
LSK cells were co-cultured with freshly prepared OBs or with OBs cultured in complete medium for 1, 2, or 3 weeks prior to co-culture with LSK cells. After 7 days of co-culture, hematopoietic cells were assayed as follows: (*A*) total number of hematopoietic cells in culture; (*B*) total number of CFUs produced in culture; (*C*) percentage of cells expressing Lin-Sca1+ cell surface markers; and (*D*) plating efficiency of clonogenic cells (number of CFUs/number of cells assayed × 100). Results are reported as box plots representing the range of data points, with the line representing the average. Error bars represent the standard deviations associated with the mean. Data points more than 2 standard deviations from the mean are identified on the plot but were used in statistical determinations. For all hematopoietic parameters analyzed, results from freshly prepared OB cultures were superior to those from OB cultures previously cultured for 1, 2, or 3 weeks prior to co-culture.

To determine the effects of OB culture duration on HPC properties, we co-cultured LSK cells for 1 week with freshly prepared OBs (*n* = 7–9) or OBs cultured for various durations (1 week, 2 weeks, or 3 weeks, n = 2 for all groups), in complete medium, prior to co-culture with LSK cells. Bonferroni post hoc corrections were performed because multiple comparisons were made. As shown in Fig. 2, freshly prepared OBs were significantly better at enhancing all HPC properties than OBs cultured for any duration prior to co-culture with LSK cells, with the exception of 1-week OB plating efficiency.

Total hematopoietic cell number was significantly higher in cultures containing fresh OBs (3.5 ± 0.3 × 10^6^) compared with OBs cultured for 1 week (1.2 ± 0.4 × 10^6^, *p* = .05), 2 weeks (1.0 ± 0.5 × 10^6^, *p* = .03), or 3 weeks (0.8 ± 0.3 × 10^6^, *p* = .02) prior to LSK seeding (Fig. 2*A*). Total CFU number was also significantly elevated in fresh OB co-cultures (34,300 ± 4000) compared with OBs cultured for 1 week (9200 ± 1500, *p* = .04), 2 weeks (3300 ± 600, *p* = .01), or 3 weeks (1800 ± 800, *p* = .007) prior to co-culturing with LSK cells (Fig. 2B). Further, the percentage of Lin-Sca1+ cells was also significantly higher in fresh OB co-cultures (29.5 ± 3.6%) compared with OBs cultured for 1 week (5.3 ± 0.9, *p* = .02), 2 weeks (0.9 ± 0.2, *p* = .008), or 3 weeks (1.0 ± 0.2, *p* = .008) prior to LSK seeding (Fig. 2C). Finally, the plating efficiency was significantly increased in fresh OB co-cultures (22.9 ± 1.6%) relative to OBs cultured for 2 weeks (9.5 ± 3.5%, *p* = .02) or 3 weeks (5.5 ± 0.5, *p* = .003) but not 1 week (17.3 ± 3.3%, *p* = .86) prior to LSK seeding (Fig. 2*D*). It should be noted that, as shown in Fig. 1, when LSK cells were cultured without OBs, hematopoietic parameters tested were significantly lower than those observed with fresh OBs; however, no significant differences were detected when comparing LSK cells cultured alone to LSK cells co-cultured with 1-week OBs.

**Fig. 3:**
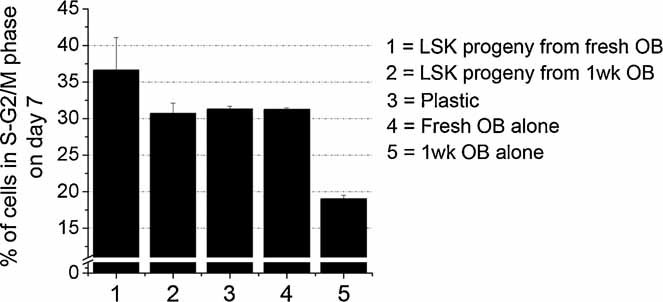
LSK cells were cultured alone or were co-cultured with freshly prepared OBs or with OBs cultured for 1 week. After 7 days of co-culture, hematopoietic cells were removed by washing and cell cycle status was assessed by staining with propidium iodide. No differences in the percentage of hematopoietic cells in the S/G2 + M phases of cell cycle were observed. In parallel studies, fresh and 1-week OBs were cultured alone and OB cell cycle status determined as above. As would be expected, a higher percentage of fresh OBs were in the active phases of cell cycle than those cultured for 1 week. In data not shown, we also analyzed both hematopoietic and OB cells for the expression of annexin V. In all cases, less than 1% of the cells were annexin V positive, suggesting that apoptosis was not detected.

Next, we conducted additional studies to examine the apoptotic and cell cycle status of hematopoietic cells following co-culture with OBs. Because the hematopoietic cell proliferation was markedly and similarly reduced in 1-, 2-, and 3-week OB cultures relative to fresh OB cultures, we chose to examine fresh and 1-week OB cultures only. The cell cycle data for hematopoietic cells cultured with fresh OBs, 1-week OBs, or cultured alone (plastic) is shown in Fig. 3 (*n* = 3/group). No significant differences were detected in the percentage of cycling cells (S-G2/M phases). Also seen in Fig. 3 is the cell cycle status of the fresh and 1-week OBs that were cultured alone. As would be expected, freshly seeded OB cultures contained significantly more cells in the active phases of cell cycle than 1-week OB cultures, which generally obtain confluence by day 5. With regard to apoptosis, in all of the cultures tested (same as those tested for cell cycle status) less than 1% of cells (hematopoietic or OB) were undergoing apoptosis defined as annexin V+ PI- cells (data not shown). Thus, loss of hematopoietic function observed in cultures where OBs have been seeded for 1 or more weeks does not appear to be due to alterations in cell cycle status or apoptosis.

### Effects of OB cell number on HPC proliferation, CFU expansion, maintenance of primitive phenotype, and plating efficiency

Because all OBs were seeded at the same original concentration, cultures containing freshly prepared OBs had fewer total OBs than did the other cultures, both at initiation and possibly throughout the co-culture period. Therefore, it is formally possible that OB cell number alone could explain the data shown in Fig. 2, due to a crowding effect. Here we show data from separate studies to address this issue.

In parallel cultures, 1-week (*n* = 4), 2-week (*n* = 4–5), and 3-week (*n* = 6) OB cultures were trypsinized, and these cells were seeded again at the same concentration as freshly prepared OBs (*n* = 7–9). All groups were co-cultured with LSK cells for 1 week and LSK properties were determined. Proliferation and CFU data from these studies showed the same trend described in Fig. 2 (data not shown): freshly prepared OBs were superior to trypsinized OBs cultured for 1, 2, or 3 weeks for all hematopoietic parameters tested. With respect to the percentage of Lin-Sca1+ cells and the plating efficiency, the trends are the same as those seen in Fig. 2. However, significant differences were detected only between freshly prepared OBs and OBs trypsinized after 3 weeks for plating efficiency (data not shown).

### LSK cells co-cultured with fresh OBs have a higher repopulating potential than LSK cells co-cultured with 3-week OBs

**Fig. 4:**
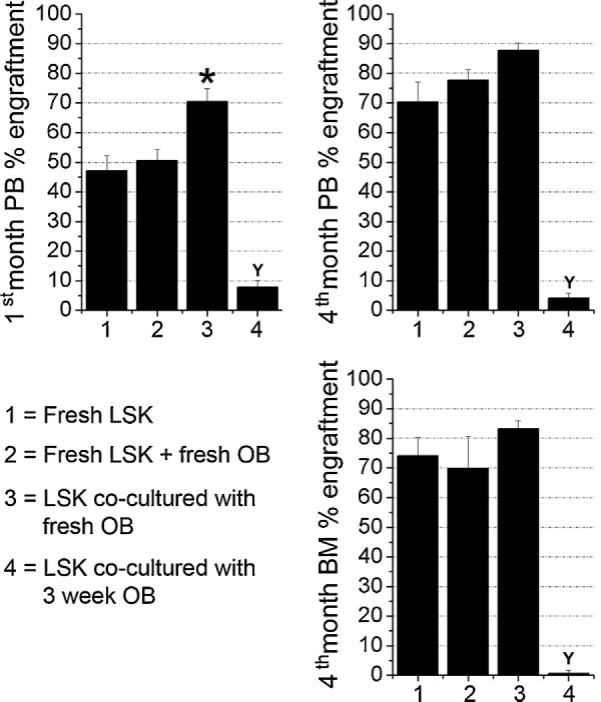
Repopulating potential of freshly isolated LSK cells in the presence and absence of fresh OBs and in vitro expanded LSK cells for 5 days in co-cultures of fresh OBs and 3-week OB cultures. Data are from one experiment and 5 mice per group were analyzed. Chimerism in the peripheral blood at 1 and 4 months post-transplantation and in the marrow at 4 months shows significantly better engraftment from LSK cells expanded in fresh OB cultures than with LSK cells expanded in 3-week OB cultures. Data are presented as the mean ± the standard deviation. *Indicates, statistically significant differences (*p* < .05) compared to fresh LSK (group 1). ^Y^ Indicates statistically significant differences (p < .05) compared with LSK co-cultured with fresh OBs (group 3).

We previously demonstrated that LSK cells harvested after 10 days of co-culture with fresh OBs were capable of repopulating the marrow of lethally irradiated recipients at levels comparable to those obtained with freshly isolated LSK cells (when expansion-equivalent numbers of cells are used) and of sustaining high and comparable levels of chimerism in secondary recipients.(54) Here, we performed similar studies where LSK cells were co-cultured with fresh or 3-week OBs for 5 days and the contents of each well were harvested and injected along with 100,000 competitor cells into lethally irradiated recipient mice. As a control we transplanted freshly isolated LSK cells with 100,000 competitor cells. For an additional control we transplanted freshly isolated LSK cells with competitor cells, as well as the progeny of 40,000 freshly isolated OBs maintained in culture for 5 days. This latter control was used to show whether the transplantation of OBs along with LSK cells facilitates engraftment. As illustrated in Fig. 4, transplantation of OBs along with LSK cells did not affect the percentage engraftment as measured in monthly peripheral blood analyses and in 4-month bone marrow cell analyses (group 1 vs. group 2). With respect to the long-term repopulating potential of LSK cells co-cultured with fresh versus 3-week OBs, data in Fig. 4 show the significant reduction in chimerism when LSK cells were co-cultured with 3-week OB compared with fresh OBs (*p* < .000002 for all monthly blood analyses and bone marrow analyses). Further, our data also suggest that co-culture with OBs enhances the expansion of a group of short-term repopulating cells, as evidenced by the significantly higher level of chimerism at 1 month post-transplantation in the group of mice receiving LSK cells co-cultured with fresh OBs (*p* = .009, group 1 vs. group 3). This observation was previously noted in similar experiments reported by our group.(54)

### Characterization of gene expression and calcium deposition in OB cultures

Freshly isolated OBs are likely composed of cells of different developmental stages, and it is generally thought that with longer culture duration and with supplementation of ascorbic acid and β-glycerophosphate, OBs become more differentiated. To examine the effects of culture conditions on OB maturation, we examined expression of genes for Runx2, alkaline phosphatase, type I collagen, osteopontin, and osteocalcin. We also examined calcium deposition as a marker for mineralization. All parameters were measured in parallel cultures when LSK seeding would occur.

**Fig. 5:**
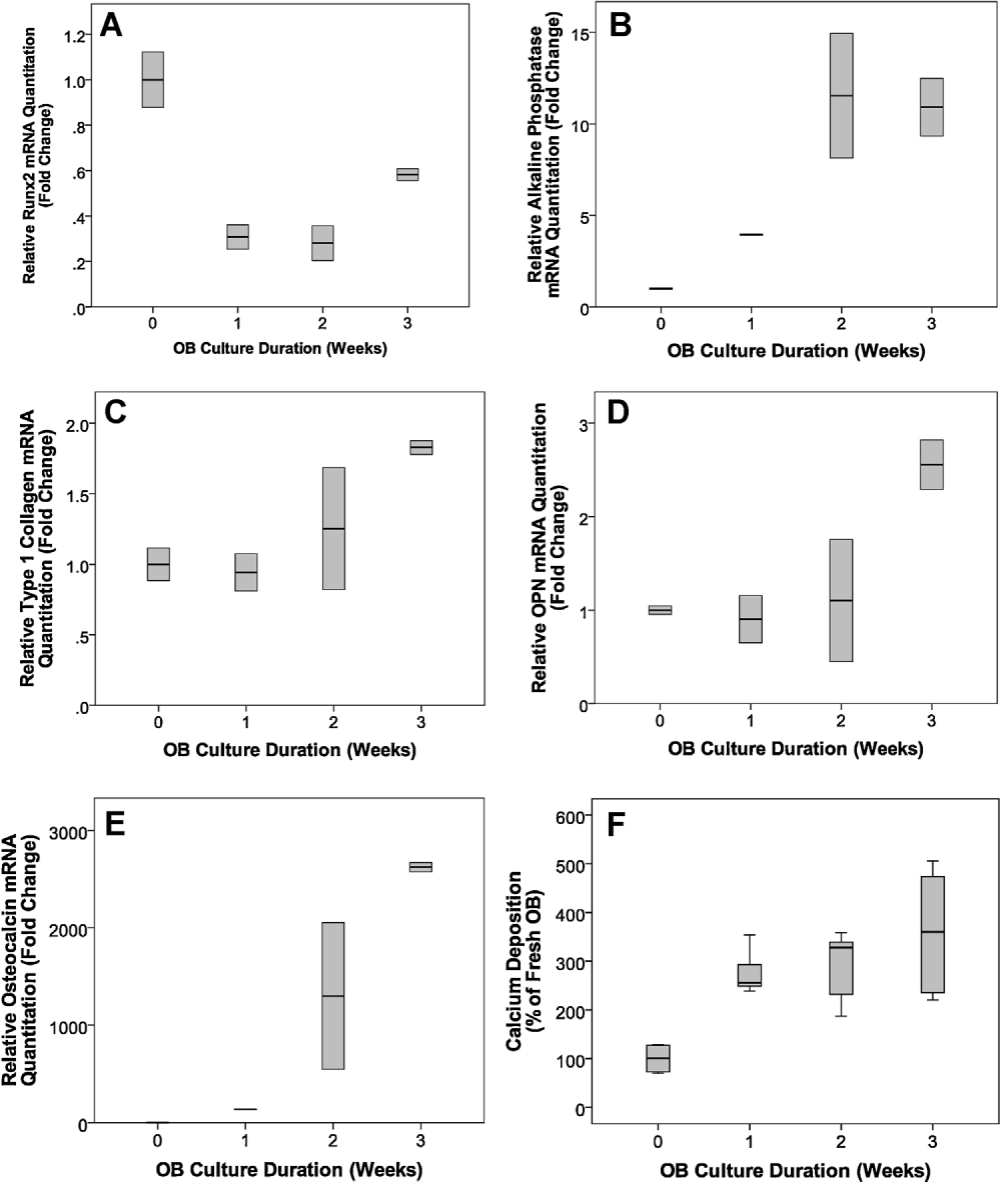
Characterization of mRNA expression and calcium deposition in OB cultures (7 days following experiment initiation) from freshly prepared OBs or OBs cultured for 1, 2, or 3 weeks prior to experiment initiation. It should be noted that for all (*A*–*E*) gene expression data and (*F*) calcium deposition data, levels were normalized to levels measured in cultures of fresh OBs. Results are reported as box plots representing the range of data points, with the line representing the average. Error bars represent the standard deviations associated with the mean (all error bars are graphed and in some cases the error bars are within the box plots). There were no data points more than 2 standard deviations from the mean. (*A*) Runx2 expression was highest in freshly isolated OBs and declined with culture duration. (*B*) Alkaline phosphatase expression increased with culture duration, peaking at week 2, and this level was maintained in week 3 cultures. As expected, expression of (*C*) type I collagen, (*D*) osteopontin, and (*E*) osteocalcin, as well as (*F*) calcium deposition, increased as OB culture duration increased.

Runx2 is a transcription factor that is highly expressed early in OB maturation and is reduced as OB differentiation progresses. As detailed in Fig. 5*A*, Runx2 expression was highest in fresh OB cultures. Alkaline phosphatase is the major enzyme expressed by OB. Figure 5*B* illustrates that alkaline phosphatase expression increased up to 2 weeks of culture duration then maintained this high level at 3 weeks. Type I collagen is the main collagen produced by OBs. Osteopontin and osteocalcin are two of the main noncollagenous bone matrix proteins produced by OBs. Figure 5*C-E* shows that as OB culture duration increases, type I collagen, osteopontin, and osteocalcin expression all increase, which is consistent with increasing OB maturation. It is important to note the striking increase in osteocalcin expression. It is well known that osteocalcin expression turns on late in OB differentiation, which is consistent with maturation increasing relative to increasing OB culture duration in our studies.

Finally, we confirmed OB function by measuring calcium deposition. It is well known that addition of ascorbic acid and β-glycerophosphate to OB cultures enhances OB differentiation and mineralization.(55) Therefore, it is not surprising that calcium deposition was increased when OB culture duration increased (Fig. 5*F*).

Next, we performed a linear regression using analysis of variance model (ANOVA) to determine whether significant associations were found between independent and dependent variables. Pearson's correlation coefficient was used to determine *R*^*2*^ values. First, we wanted to determine whether significant associations were found between OB culture duration and OB gene expression/calcium deposition. These analyses showed that OB culture duration is significantly associated with expression of alkaline phosphatase (*p* = .007, *R*^*2*^ = .733), type I collagen (*p* = .04, *R*^*2*^ = 0.549), osteopontin (*p* = .05, *R*^*2*^ = 0.494), osteocalcin (*p* = .002, *R*^*2*^= 0.808), and calcium deposition (*p* < .001, *R*^*2*^= 0.545). Statistical significance was not achieved for associations between OB culture duration and Runx2 expression.

Second, we wanted to determine whether significant associations existed between HPC properties and OB gene expression/calcium deposition. The data show that Runx2 is significantly associated with hematopoietic cell number (*p*= .03, *R*^*2*^= 0.828) and the percentage of Lin-Sca1+ cells sustained for 7 days in co-cultures of OB and LSK cells (*p* = .04, *R*^*2*^ = 0.789). While the association did not reach significance between Runx2 and number of clonogenic cells (*p* = .06, *R*^*2*^ = 0.734) it was by a small margin, but significance was reached between Runx2 expression and chimerism in repopulation studies (*p* = .02, *R*^*2*^ = 0.916). The data also show that chimerism in repopulation studies is significantly and inversely associated with OB expression of alkaline phosphatase (*p* = .007, *R*^*2*^ = 0.974), type I collagen (*p* = .009, *R*^*2*^ = 0.964), osteopontin (*p* = .03, *R*^*2*^ = 0.897), and osteocalcin (*p* = .003, *R*^*2*^ = 0.988). Further, these analyses showed that calcium deposition is inversely associated with hematopoietic cell number (*p* = .002, *R*^*2*^ = 0.713), number of clonogenic cells (*p* = .01, *R*^*2*^ = 0.561), percentage of Lin-Sca1+ cells sustained for 7 days in co-cultures of OB and LSK cells (*p* = .007, *R*^*2*^= 0.664), and chimerism in repopulation studies (*p* < .001, *R*^*2*^ = 0.962). While the association did not reach significance for plating efficiency, it was by a small margin (*p* = .08, *R*^*2*^ = 0.342).

In all of the above analyses the effects of OB properties on hematopoietic enhancing activity was examined. That said, it is possible that co-culture of hematopoietic cells with OBs may alter OB maturation, which in turn can impact hematopoietic cells. We therefore cultured OBs in the presence or absence of LSK cells for 1 week, removed LSK cells by gentle washing as detailed above, and isolated mRNA from OBs for real-time PCR analysis. For these studies we examined the same gene products examined above (Runx2, alkaline phosphatase, type I collagen, osteopontin, osteocalcin), as well as Jagged1 and Jagged2. Jagged1 and Jagged2 are both Notch ligands that are highly expressed on OBs. Notch signaling has been shown by us(54) and others(56) as being important in OB-induced hematopoietic-enhancing activity. There were no significant differences detected for any of these genes in OBs cultured alone or in the presence of LSK cells (data not shown). Similarly, in previous studies, we did not detect differences in OB morphology or cell number when comparing OBs cultured alone to OBs co-cultured with LSK cells.(57) Taken together, these data appear to suggest that OB lineage cells responsible for the hematopoiesis-enhancing activity are present in the culture de novo.

## Discussion

Our findings illustrate that OBs sustain HPC survival and proliferation and that this support is dependent on the stage of maturation and differentiation of the OBs. Using multiple in vitro functional assays and phenotypic assessment of cultured hematopoietic cells, we demonstrated that fresh OBs supported significantly higher HPC expansion compared with what was observed with LSK cells cultured alone. This OB support of hematopoiesis declined when OBs were cultured for increasing durations. That cultured OBs matured and differentiated during a period of 3 weeks was shown by both functional and molecular determinations. Importantly, in vivo transplantation studies corroborated our in vitro results with respect to the relationship between HPC support and stage of OB maturation. Furthermore, our in vitro data are consistent with, and relevant to, aging. It is well understood that with age, bone mass is lost, OB number is reduced, and HSC/HPC function also declines.(58–62) Our previously published histomorphometric mouse bone data also support the reduction in bone volume and OB number with age.(63) Indeed, we reported in control mice an approximately 2.5-fold reduction in bone volume and a greater than 10-fold reduction in OB number in 9-month-old mice compared with that in 6-week-old mice. In our studies presented here, extending OB culture duration allows for OB maturation; therefore, 3-week OB cultures contain fewer early-stage or immature OB lineage cells than that observed in fresh OB cultures. Thus, our in vitro cultures mimic the in vivo loss of early stage OB lineage cells seen in aging. Furthermore, in both our in vitro studies and in aging, HSC/HPC function is reduced.

With respect to our in vitro studies, fresh OBs promoted a significantly higher level of HPC expansion (total cell number produced in culture), CFU production, and maintenance of Lin-Sca1+ cells in culture than did OBs cultured for 1, 2, or 3 weeks. Given that c-kit expression (CD117) is quickly downregulated through the internalization of the receptor in cultures supplemented with exogenous SCF,(50–52) we did not use CD117 to track the phenotypic makeup of cultured cells (Supplemental Fig. 1). Still, evaluation of Lin-Sca1+ cells is a valid approach for measuring the maintenance of primitive HPCs in culture. Of note, when LSK cells were co-cultured with OBs alone (no additional cytokines added) a similar trend in expansion of hematopoietic cell number was observed, albeit to a much lesser extent, which limited our ability to conduct further analyses.

Interestingly, with respect to cytokine-supplemented cultures, a slightly different picture was seen with plating efficiency. For this parameter, no difference was detected between freshly prepared OBs and those cultured for 1 week. However, significant differences were detected between freshly prepared OBs and those cultured for either 2 or 3 weeks prior to LSK seeding. The lack of difference in plating efficiency observed between freshly prepared OBs and those cultured for 1 week is likely owing to the fact that plating efficiency may not change as dramatically and quickly as the other parameters reported. No differences in cell cycle status or apoptosis were observed in hematopoietic cells co-cultured with fresh OBs or with 1-week OBs. Thus, the loss of hematopoietic function observed in cultures where OBs have been seeded for 1 or more weeks does not appear to be due to alterations in cell cycle status or apoptosis, but rather due to intrinsic differences in fresh versus cultured OBs.

In our model system, a confluent monolayer of freshly prepared OBs is observed within 2 to 3 days. Only in the fresh OB culture are LSK cells present for few days in a nonconfluent environment, so although cell number should be theoretically similar in all cultures by day 7 (because of contact inhibition), it is formally possible that the higher cell number in the 1-, 2-, and 3-week cultures immediately after seeding with LSK cells could have accounted for the decline in LSK properties because of a cell “crowding phenomenon” and possible limited access to nutrients. We addressed this possibility by reseeding the more mature OBs grown for 1, 2, and 3 weeks at identical starting concentrations and assessing HPC properties. These studies recapitulated all of the data obtained in the main arm of this study, including the plating efficiency results showing that our findings were not an artifact of crowding or nutrient consumption, but were instead related directly to OB culture duration and presumably the stage/maturation of the OBs in culture.

We then sought to confirm our in vitro findings using the “gold standard” in vivo reconstitution studies. Given that we have recently shown that LSK cells maintained in co-cultures with fresh OBs for 10 days maintain their in vivo repopulating potential,(54) we examined, in a similar system, the ability of LSK cells co-cultured with fresh or 3-week OBs to engraft and sustain in vivo hematopoiesis. Since differences in the in vitro hematopoietic functions were highest between fresh and 3-week OBs, we chose those two co-cultures for in vivo studies. Our in vivo data corroborated in vitro results in that LSK cells maintained in culture with fresh OBs for 5 days supported a significantly higher level of chimerism than their counterparts maintained with 3-week OBs (Fig. 4). Importantly, in conducting these studies, we included control groups to determine whether engraftment was facilitated by the OBs contained within the harvested wells. In our system, OBs themselves were not responsible for enhancing engraftment; rather, in vitro expansion of LSK cells on the OBs was necessary for the enhanced engraftment. These in vivo data are significant because they provide a strong confirmation of our claim that fresh, less mature OBs support the hematopoietic function of progenitor cells better than more mature or cultured OBs.

That OBs cultured for 2 or 3 weeks in our system represent differentiated or mature osteoblastic lineage cells as previously speculated(64) was confirmed by examining well-established markers. As would be expected from a maturation-associated pattern of expression or loss of expression of OB-specific markers, we observed a time-dependent loss of Runx2, a transcription factor required for OB differentiation; an increase in the expression levels of type I collagen, osteopontin, osteocalcin; and an increase in the main osteoblastic enzyme, alkaline phosphatase. Consistent with these patterns, cultured OBs deposited greater amounts of calcium in a time-dependent manner, as previously shown.(41) Calcium has been suggested to be highly influential in promoting the survival and differentiation of HSCs in the niche.(65) We believe that the inverse relationship detailed here does not stand in contrast to this principle, because calcium deposition in our studies was used as a marker for OB mineralization(66,67) and does not reflect the amount of bioavailable calcium. Coupled with in vitro and in vivo functional data, these results show that early-stage OBs are superior in enhancing or maintaining hematopoietic progenitor and stem function, including the marrow-repopulating potential of these cells.

At face value, our data do not show whether the hematopoietic-supportive OB lineage cell is in the culture de novo or whether culture with LSK cells alters OB differentiation such that this alteration is responsible for the hematopoiesis-enhancing activity. To examine this possibility, we cultured OBs alone and in the presence of LSK cells for 1 week and analyzed OB mRNA expression of Runx2, alkaline phosphatase, type I collagen, osteopontin, osteocalcin, Jagged1, and Jagged2. No differences were observed, suggesting that LSK cells themselves do not appear to alter OB differentiation. These data suggest, therefore, that the hematopoietic-supportive OB lineage cell is most likely present de novo and not modified with LSK culture.

Overall, these data suggest a dynamic relationship between hematopoiesis and the state of turnover of OBs in the hematopoietic niche. Based on our results, increasing the number of early stage OBs in the hematopoietic niche through pharmacologic or chemical interventions may be an effective technique for optimizing the therapeutic benefit of stem cell transplantation in cancer patients, as previously suggested.(68–70) Furthermore, our results begin to explain the possible relationship between aging and hematopoiesis as a result of OB activity. This activity is substantially increased in times of high bone turnover and remodeling as observed in the pediatric population, which is mirrored by increased levels of hematopoiesis. As bone ages and red marrow diminishes, so, too, do bone turnover and OB activity diminish. This concept has been suggested previously(71) and would warrant further investigation. Moreover, other pathologies in which OB activity is either accelerated or diminished may also bear a correlation to hematopoietic abnormalities. In conclusion, while OBs in general are known to exert a regulatory effect on HSCs in the hematopoietic niche, our results provide evidence that OBs in earlier stages of differentiation may be primarily responsible for these activities.
