# Supplementary figures and images for: Impact of Maturational Status on the Ability of Osteoblasts to Enhance the Hematopoietic Function of Stem and Progenitor Cells
Source: J Bone Miner Res. 2010 Nov 23;26(5):1111–21. doi: 10.1002/jbmr.302 (PMC3179304; doi:10.1002/jbmr.302)

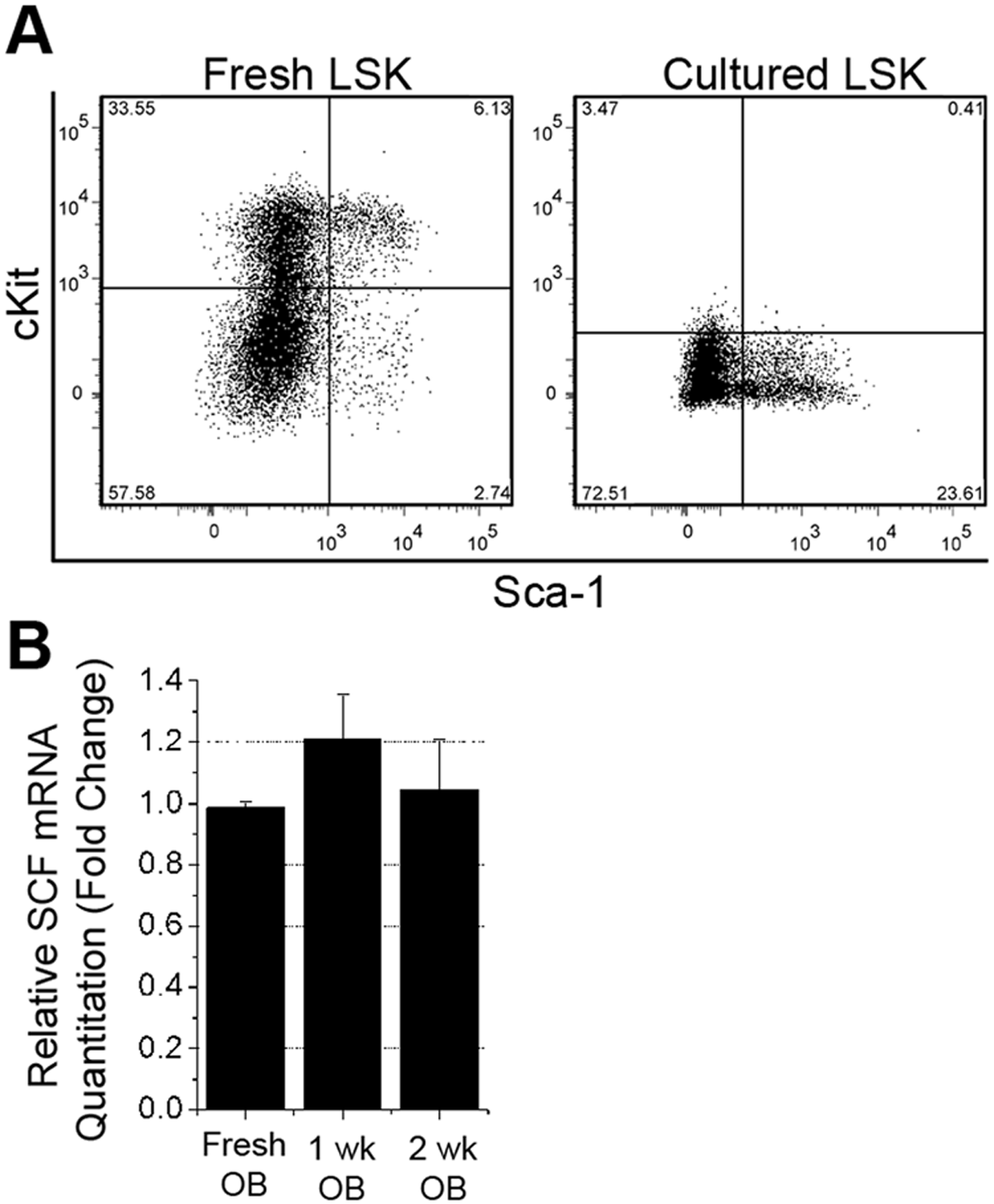

Supplement: Supplementary file 1 [file jbmr0026-1111-SD1.tif]
